# Filtration Parameters Influencing Circulating Tumor Cell Enrichment from Whole Blood

**DOI:** 10.1371/journal.pone.0061774

**Published:** 2013-04-26

**Authors:** Frank A. W. Coumans, Guus van Dalum, Markus Beck, Leon W. M. M. Terstappen

**Affiliations:** Medical Cell BioPhysics, MIRA Institute, University of Twente, Enschede, The Netherlands; University of Arizona, United States of America

## Abstract

Filtration can achieve circulating tumor cell (CTC) enrichment from blood. Key parameters such as flow-rate, applied pressure, and fixation, vary largely between assays and their influence is not well understood. Here, we used a filtration system, to monitor these parameters and determine their relationships. Whole blood, or its components, with and without spiked tumor cells were filtered through track-etched filters. We characterize cells passing through filter pores by their apparent viscosity; the viscosity of a fluid that would pass with the same flow. We measured a ratio of 5·10^4^∶10^2^∶1 for the apparent viscosities of 15 µm diameter MDA-231 cells, 10 µm white cells and 90 fl red cells passing through a 5 µm pore. Fixation increases the pressure needed to pass cells through 8 µm pores 25-fold and halves the recovery of spiked tumor cells. Filtration should be performed on unfixed samples at a pressure of ∼10 mbar for a 1 cm^2^ track-etched filter with 5 µm pores. At this pressure MDA-231 cells move through the filter in 1 hour. If fixation is needed for sample preservation, a gentle fixative should be selected. The difference in apparent viscosity between CTC and blood cells is key in optimizing recovery of CTC.

## Introduction

Circulating tumor cells (CTC) are cancer cells disseminated into the blood from primary or metastatic sites. Clinical trials have shown that the presence of CTC is predictive of survival in several types of cancer, including breast, prostate, colon, gastric, small and non-small cell lung carcinoma and melanoma [1]–[7]. Because the typical CTC concentration is 1 CTC in 1 mL of blood [8] (compare to 5·10^6^ white cells and 5·10^9^ red cells), enrichment of CTC is the first step in most CTC enumeration approaches. Selective CTC enrichment is achieved either by positive selection, targeting antigens on the cell surface of the CTC not expressed by blood cells, or by selective depletion of the blood cells targeting antigens not expressed on CTC [9]–[15]. The downside of using antibody mediated positive enrichment is that cells with low or no expression of the antigen are lost. Antigen expression independent techniques could select CTC based on the physical differences between tumor and blood cells, for example: stiffness [16], density [17], size by a filter membrane [18]–[28] or other filter type [29], [30]. Recent filtration methods [20]–[28] report much improved recoveries compared to early methods [18], [19]. However, large unexplained differences in sample fixation, sample dilutions, flow rates and pressures across the filters exist between approaches as summarized in table 1. We expect that these parameters affect whether a large cell passes through a small pore as they influence red and white blood cells [31]–[33], and therefore it is not feasible that all parameter combinations in table 1 are optimal. Here we investigate which of these parameters are indeed important for enrichment of CTC using filtration techniques. After identification of the important parameters for CTC enrichment the filter characteristics that are optimal for CTC enrichment were investigated in an accompanying paper[34].

**Table 1:** Filtration methods and conditions.

Reference	Pressure (mbar)	Pore size (µm)	Fixation	Sample dilution
Zheng 2011 [26]	35	7*	none	1∶10
Zheng 2007 [23]	35	10	PFA‡	1∶10
Tan 2010 [30]	50	5*	none	none
Vona 2000 [28]	300	8	PFA‡	1∶10^§^
Desitter 2011 [22]	700^†^	6.5	none	1∶8^§^
	700^†^	7.5	PFA‡	1∶7^§^
Kahn 2004 [21]	N/A	8	50% EtOH	5∶4^§^

* estimated pore size from 3D filter structure, ^†^pressure at start of filtration,

‡ paraformaldehyde, ^§^red blood cells lysed/removed.

## Materials and Methods

### Blood samples

Healthy volunteers aged 20–55 gave written informed consent before donating blood, the study protocol was approved by the METC ethics board (Enschede, The Netherlands). Blood from EDTA vacutainers (BD, Franklin Lakes, NJ, USA) was processed within 12 hours after draw. Unless otherwise noted, each data point represents the average of three repeat measurements. For each repeat we used blood from a different donor, but a whole experiment was done with the same three donors.

### Cell culture and cell staining

Spiking experiments were performed with cells from the prostate carcinoma cell line PC3-9, a sub-clone of the PC3 cell line [35] kindly provided by Immunicon (Huntingdon valley, PA, USA) and the breast carcinoma cell lines SKBR3 and MDA-231 obtained from ATCC (Manassa, VA, USA). PC3-9 cells were cultured using RPMI (Sigma, St. Louis, MO, USA) while the SKBR3 and MDA-231 cells were cultured in Dulbecco's Modified Eagle Medium (Sigma). Culture media were supplemented with 10% fetal bovine serum (Gibco, Invitrogen, Carlsbad, CA, USA), 1% penicillin-streptomycin (Gibco) and 1% L-Glutamin (Sigma). PC3-9 were stained with CellTracker Green Bodipy, MDA-231 with CellTracker Orange CMTMR (both Invitrogen) and SKBR-3 with both stains. Cells were incubated in culture media for 24 hours at 37°C with 50 µM CellTracker Green and/or 5 µM CellTracker Orange prior to harvesting with 0.05% trypsin (Gibco). While still in the filter holder, the filter was washed with ethanol to fix the cells to the filter, in a series of increasing concentration from 70 to 100%. The filter was dried in vacuum followed by staining of nuclei with 8µM Hoechst 33342 (Invitrogen).

### Filtration setup

**Figure 1:**
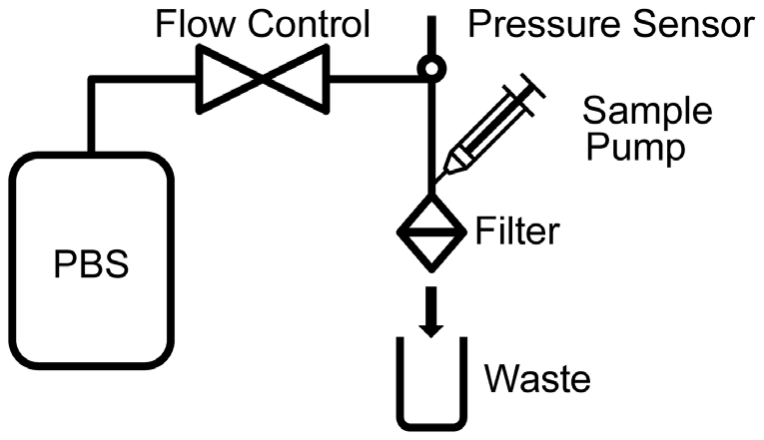
Setup for filtration. The setup allows for control of either pressure, flow rate or sample dilution factor while monitoring the other two parameters.

A filtration setup was constructed to allow simultaneous measurement of pressure and control of flow rate, figure 1. The flow through the filter consists of two parts, the sample flow and a PBS (phosphate buffered saline) flow. If desired, the PBS flow can be used to dilute the sample. Dilution of 1∶*x* means that 1 part sample is diluted in *x*-1 parts of PBS. For the sample flow, a sample is loaded into a 1 mL or 50 mL syringe (Plastipak, BD, Franklin Lakes, NJ, USA) with a 21 gauge needle (Microlance 3, BD) and placed onto a NE-1000 syringe pump (New Era Pump Systems, Farmingdale, NY, USA). The PBS flow originates from a stainless steel tank (Alloy products, Waukesha, WI, USA) pressurized with 2 bar N_2_. To reduce uptake of N_2_ into the PBS, the PBS is contained inside a plastic bladder inside a water filled tank. PBS is filtered by an inline 0.2 µm filter (mini Kleenpak, Pall, Mijdrecht, The Netherlands) and the flow rate is controlled by a flow sensor (CoriFlow, Bronkhorst, Veenendaal, Netherlands). The pressure difference across the filter is measured by a 0–300 mbar pressure sensor (PR-41X, Keller, Winterthur, Switzerland). During filtration, the pressure increases until a stable situation is reached or pressure exceeds 300 mbar. The stable pressure was recorded.

### Filters

Track-etched filters with pore sizes of 5 µm and 8 µm pore size, a diameter of 13 mm and a thickness of 6–11 µm were used (Nucleopore, Whatman, GE, Maidstone, UK). The number of pores on a track-etched filter were counted with a bright field image of 1 mm^2^ of filter area using a fluorescent microscope with 4×NA = 0.13 objective (E-400, Nikon, Melville, NY, USA). The 5 µm pore size filters contained 3.5·10^5^ pores and the 8 µm contained 9.2·10^4^ pores in a filter surface of 102 mm^2^. Inspection of the filters by bright field microscopy indicates that (1) the pores are distributed randomly, that (2) 3–10% of the pores are so closely spaced that one 15 µm diameter cell may block several pores, and that (3) 1–2% of pores consist of merged holes. The pores constitute approximately 5–7% of the filter area. Since filter properties vary between different lots, only one lot of each pore size was used. The filters were mounted in plastic filter holders (Swinney, Pall). Before use, a filter was placed in a holder, primed with PBS and placed in ∼50 mbar vacuum for 30 minutes, which displaced trapped air form the filter pores, but did not evaporate more than 10% of the PBS.

### Relation between number of pores, pressure, and flow rate

The relation between pressure and flow rate for a filter was determined by increasing the flow rate from 0–800 mL/h in steps of 50 mL/h and recording flow rate and pressure. To determine the impact of the number of pores, we covered parts of the 5 and 8 µm track-etched filters with different aperture plates leaving 2, 4, 8, 16, 32 or 57 mm^2^ of the filter area open. Viscosity of PBS was assumed to be equal to water [36], 0.9 mPa·s.

### Impact of sample dilution on pressure

To determine the impact of sample dilution on the pressure difference, we filtered 1 mL of whole blood through a 5 µm track-etched filter with dilutions of 1∶1, 1∶4 and 1∶16 and total flow rates of 50, 200 and 800 mL/h. Dilutions were set at 1∶4, and sample flow rate to 25 mL/h for all other experiments.

### Filtration of blood fractions, culture cells and beads

Major components of whole blood include white blood cells (WBC), red blood cells (RBC) and serum. Blood was split into fractions by means of centrifugation. A 10 mL tube of blood was centrifuged at 300×*g* for 10 minutes. We collected the top 1 mL of serum, the bottom 1 mL of red blood cells as well as the buffy coat layer together with 0.5 mL of serum above and 0.5 mL of red blood cells below this layer. Each fraction was reconstituted to their original concentration with PBS-1%BSA (bovine serum albumin). At a hematocrit of 60%, the 1 mL of serum was diluted with 1.5 mL of PBS-1%BSA and the 1 mL of RBC was diluted with 0.67 mL PBS-1%BSA. The buffy coat was diluted with 9 mL of PBS-1%BSA. We did not lyse the red blood cells in the buffy coat sample to prevent swelling of the WBC. As a result the sample contains RBC, but at a concentration ∼12 times lower than the RBC sample.

For culture cells, we attempted to pass 10^6^ MDA-231, PC3-9 and SKBR-3 culture cells through a 5 µm pore track-etched filter with 0.35·10^6^ pores, or three times more cells than pores. To verify the size-selectivity of the setup, we filtered a solution with 10^6^ 10 µm, 6 µm and 4 µm polystyrene beads (size calibration beads, Invitrogen) through a 5 µm filter. The filtrate was enumerated on a FACSARIA II flowcytometer (BD) using forward and side scatter signals and the beads on the filter enumerated using bright field imaging on a microscope with 4×NA = 0.13 objective.

### Whole blood cell filtration

The force pushing cells through a filter is the pressure *ΔP* across the filter times the cross section *A*_pore_ of the pore. The interaction of a cell with a pore can be modeled by describing the cell as a droplet characterized by an apparent viscosity [37], which is dependent on the cell surface tension, cell and nucleus viscosity [38], the sample temperature [39], and the deformation required to pass through a pore [40]. Below a critical pressure the surface tension of the cell membrane will counter the force pushing the cell into the filter pore. At pressures in excess of this critical pressure, the cell will move into the pore at a rate determined by the apparent viscosity. Since the apparent viscosity is cell dependent, a condition may exist where the critical pressure is exceeded for blood cells, but not for CTC. In this case filtering at a pressure below the CTC critical pressure will be an effective enrichment method. On the other hand, if the critical pressure for CTC is similar to blood cells, filtration must take place at a pressure in excess of the critical pressure for CTC to prevent clogging of the filter. At pressures in excess of the critical pressure for CTC, all cells in the sample will pass through a filter, each with a speed determined by their apparent viscosities.

For a filter with *N* pores of diameter *d*, height *h* and sufficiently low porosity (<10%), steady state laminar flow pressure difference across the filter when an incompressible fluid with viscosity µ passes through at flow rate *Q* is given by [41]–[43]: 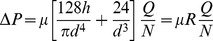
(1)
*R* is the pore resistance, derived for a pore with sharp edges. For Whatman nucelopore filters h is approximately 10 µm, d is approximately 5 or 8 µm. When h>>d equation 1 reduces to the equation for Poiseuille flow. For all our experimental conditions the Reynolds number is smaller than 100 unless a filter is nearly clogged. It is therefore sufficient to consider laminar flow conditions.

### Estimation of cell speed and apparent viscosity

Describing the cells as droplets suspended in a diluent, with the assumption that no cells are permanently retained by the filter and no fibrin aggregates that may clog the filter are present, the pressure difference must be equal for each of the components.
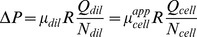
(2)


With subscripts used to indicate the contribution of the diluent (dil), cells (cell) or diluent and cells combined (total). Further *Q*_cells_ = *Q*_total_ *C*_cell_ *V*_cell_, where *C*_cell_ is the (number) concentration of cells in the sample and *V*_cell_ is the cell volume. We can now use this equation to determine the number of pores that are needed to pass the sample diluent *N*_dil_. The rest of the pores *N*_cell_ = *N*_total_−*N*_dil_ are occupied by cells passing through. The unknown apparent viscosity 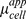 for each cell type can now be determined by filtering a single cell type with known concentration and cell volume. We assume an average of 90 fl for the red blood cell volume [44]. Other cells are assumed to be spherical with a radius of 10 µm for WBC and 15 µm for MDA-231. The cell speed *v* inside the pore is then derived according to equation 3, with *A*_pore_ the cross section of a pore. 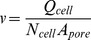
(3)


The data from the filtration of blood fractions and culture cells was used to determine apparent viscosity and cell speed for each cell type. For the WBC/RBC cell mixture in the components experiment, the speed of the WBC was estimated by subtracting the number of pores clogged by RBC as derived from the same experiment.

### Spiked samples and cell recovery for different fixations

Thirty mL of blood was collected and spiked with 300 pre-stained MDA-231, SKBR3 and PC3-9 per mL of whole blood. The total of 900 cells is expected to occupy less than 1% of the total number of pores, yet is sufficiently high for cell counting. Concentration of cells in the spiking stock was determined by counting at least 200 cells of each type on a counting chamber (Neubauer, Lauda-Königshofen, Germany). The spiked blood was split into three fractions of 10 mL. The first fraction was processed unfixed. To the second fraction the content of a 10 mL CellSave preservative vacutainer tube (Veridex, Raritan, NJ, USA) was added and incubated for 3–5 hours. The third fraction was split into 1 mL aliquots and 10±0.5 minutes prior to commencement of the filtration procedure 1 mL of a 0.8% formaldehyde solution (PFA) was added to each aliquot. For filtering, the total flow rate was set at 100 mL/h, with a 1∶4 dilution.

**Figure 2:**
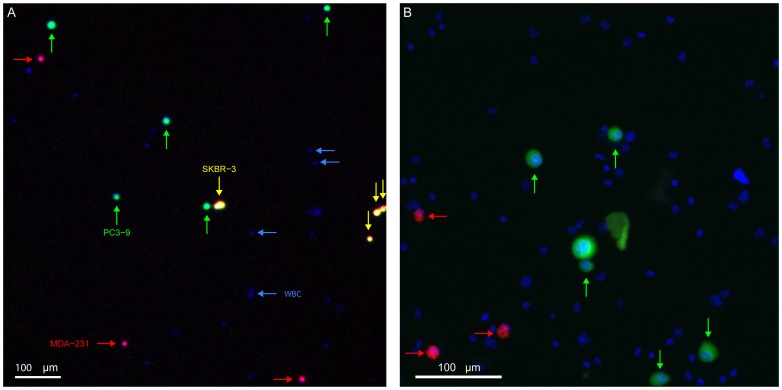
Counting cells on a track-etched filter. False color image of pre-stained culture cells and WBC on a track-etched filter using 4× (**A**) and 10× (**B**) magnification.

After filtration the filter was fixed with an ethanol series increasing up to 100% to fix the cells to the filter. The samples were enumerated by imaging the filter on a fluorescence microscope as described above. False color images (CellTracker Orange: red, CellTracker Green: green, Hoechst 33342: blue) were generated in which the PC3-9 appear light green, the MDA-231 pink and the SKBR-3 yellow, figure 2. Cells with only a nucleus are assumed to be white blood cells (blue).

## Results and Discussion

### Relation between flow rate, pressure and number of pores is predicted by the model

**Figure 3:**
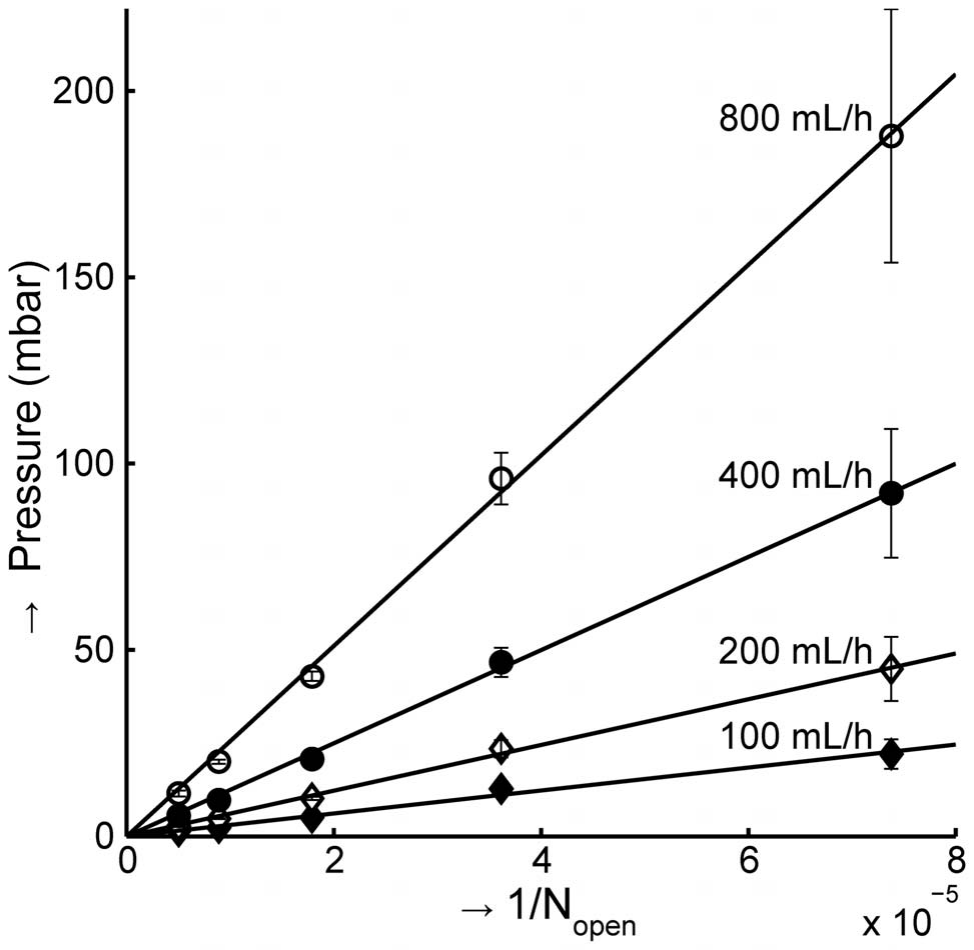
Pressure versus inverse of open pores, for different flow rates. The number of pores on a 5 µm track-etched filter was decreased by reducing the number of available filter pores with aperture plates. The fits through the data were used to estimate an average relationship between pressure (*P*; mbar), flowrate (*Q*; mL/h) and number of pores (*N*_open_) by fitting the resistance to flow (*R*); *P = µR Q*/*N*_open_. With the viscosity *µ* at 0.9·10^−3^ Pa·s, *R* is 1.28±0.02 µm^−3^.

A custom filtration setup was used to investigate the influence of pressure, sample dilution and fixation on the enrichment of large cells from whole blood by means of filtration. A linear relation between the number of pores, total flow rate and pressure across the filter was expected for laminar flow conditions, equation 1, and confirmed experimentally using phosphate buffered saline (PBS), as can be seen for different numbers of 5 µm pores in Figure 3. The resistance *R* is 1.24±0.02 µm^−3^, or 48% higher than predicted by equation 1. For the 8 µm filter, we obtain *R* = 0.26±0.18 µm^−3^, with a predicted *R* of 0.15 µm^−3^. The deviation between the theoretical *R* and the determined *R* is relatively small and could be caused by differences between real and nominal thickness of the filter or pore size, or differences in modeled versus real pore shape. The variation in *R* is attributable to experimental repeatability. We applied equation 1 to determine the number of pores that pass PBS and derive from this the number of pores that pass cells according to equation 2.

### In a blood sample the sample flow rate dominates pressure

**Figure 4:**
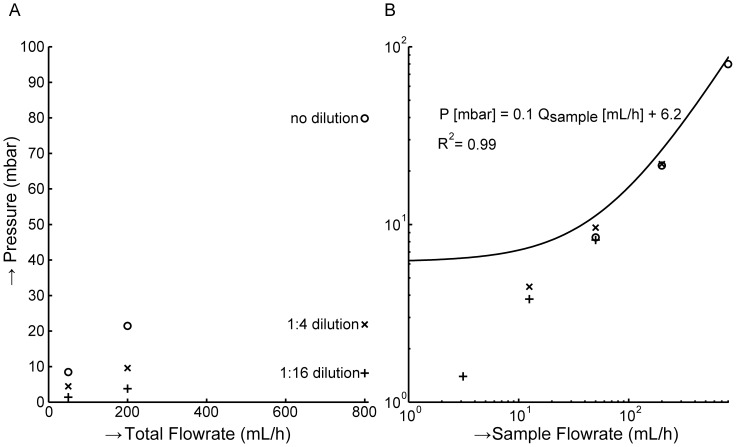
Sample flow rate determines pressure drop across filter. Whole blood was filtered through a 5 µm track-etched filter at total flow rates of 50, 200 and 800 mL/h without sample dilution and with 4 or 16× sample dilution. (**A**) Total flow rate versus pressure. In contrast with figure 3, the total flow rate is not the main factor contributing to the pressure across the filter when cells are present in the sample. (**B**) Sample flow rate versus pressure. When the pressure difference is plotted as a function of the sample flow rate, there appears to be a linear relation.

Figure 4, panel A shows the relation between total flow rate, sample dilution and pressure, averaged across 3 donors. At the same total flow rate, pressures are 10 times higher for undiluted blood than for 1∶16 diluted samples. However, relations between pressure and sample flow rate are linear for each donor (R^2^ for each donor at each dilution>0.94). Figure 4, panel B shows that the pressure is primarily determined by the sample flow rate, not the total flow rate. This demonstrates that the key parameter is the arrival rate of cells on the filter. The slope between three donors varied by ±40%, possibly because their hematocrit or white cell concentration differed [31]–[33].

Three total flow rates (50 mL/h, 200 mL/h, and 800 mL/h) were measured at a sample flow rate of 50 mL/h. In these conditions, the pressure varied from 10.5–17.2 mbar, implying that on average 15·10^3^ to 144·10^3^ pores (4–41% of the total) were needed to pass the diluent (plasma and PBS), while the rest of the pores (207·10^3^–336·10^3^) were filled with a cell passing at much lower speed. For a concentration of 5·10^9^ predominantly red blood cells per mL of sample the average speed of a cell passing through a pore is 0.9–1.5 mm/s while the average fluid flow speed is 48–78 mm/s inside the pores. The arrival rate of cells on the filter dominates pressure but a higher dilution results in a small increase of pressure, thus slightly reducing recovery. Nevertheless, we decided to set dilution at 1∶4 to clear the remaining blood from the filter holder in a timely fashion.

### RBC and WBC contribute most to the pressure

**Figure 5:**
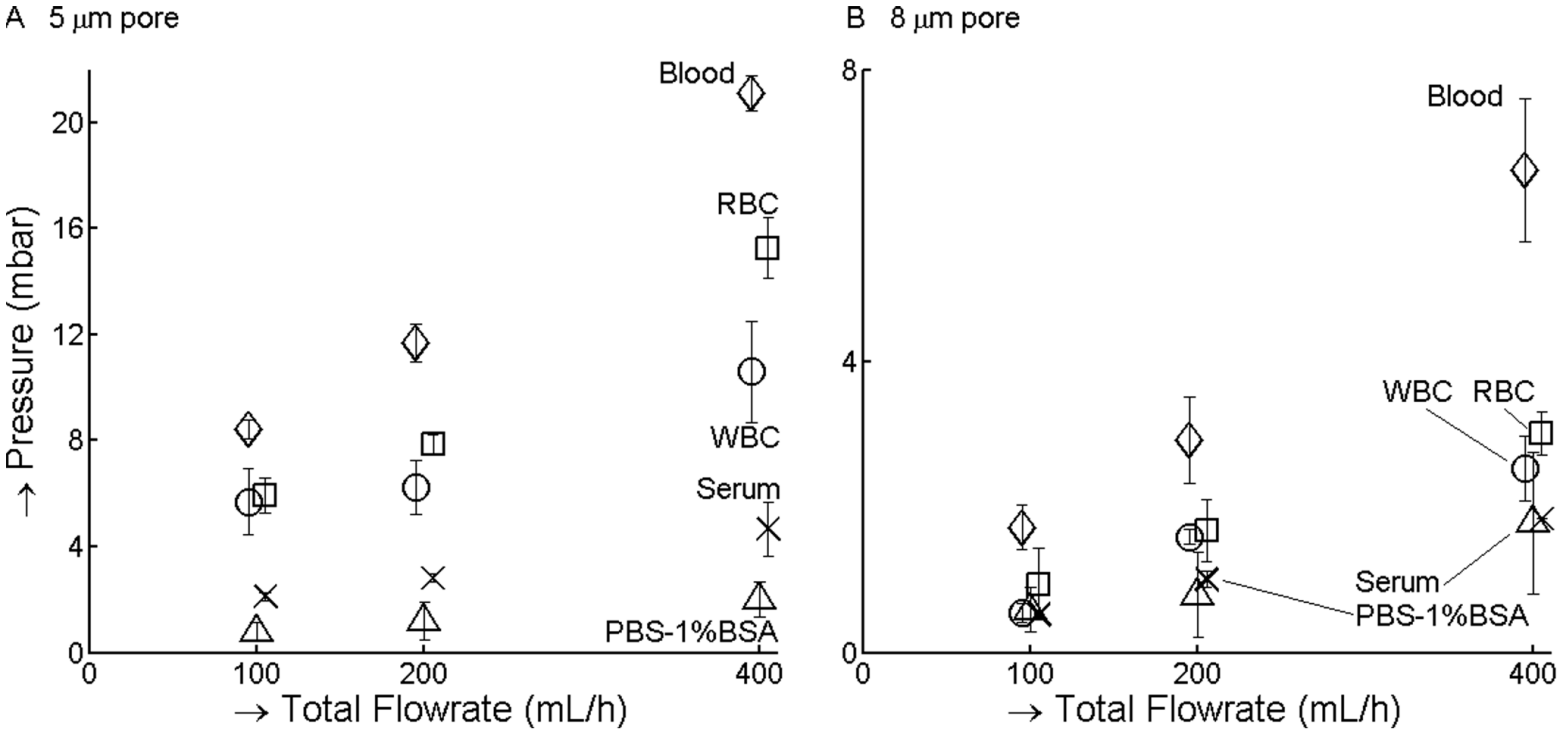
All blood components contribute to total pressure. Major blood components RBC, WBC and serum were filtered across 5 (**panel A**) and 8 (**panel B**) µm track-etched filters until pressure reached a plateau (y-axis). Whiskers show the standard deviation from three measurements. Data-points were all measured at flow rates of 100, 200 and 400 mL/hr, but were slightly offset to facilitate reading of the graph. Inspection of the filters after filtering using bright field imaging and fluorescence imaging of the Hoechst 33342 stain showed no evidence of capture of RBC, while 10^3^–10^4^ WBC were found on each filter.

To confirm that the cells are indeed the dominant factor we determined the relation between pressure and flow rate for blood components. Panel A of figure 5 shows a 1∶4 dilution with 3 different total flow rates (100, 200 and 400 mL/h). The different components tested include RBC (5·10^9^/mL), a mixture of RBC (4·10^8^/mL) and WBC (5·10^6^ WBC/mL), serum, and PBS-1%BSA solution. The serum leads to a slight increase in pressure compared with PBS-1%BSA. WBC and RBC contribute almost equally to the pressure difference, even though RBC are 1000 times more frequent.

### A 15 µm cell can easily pass through a 5 µm pore while a 6 µm bead does not pass

**Figure 6:**
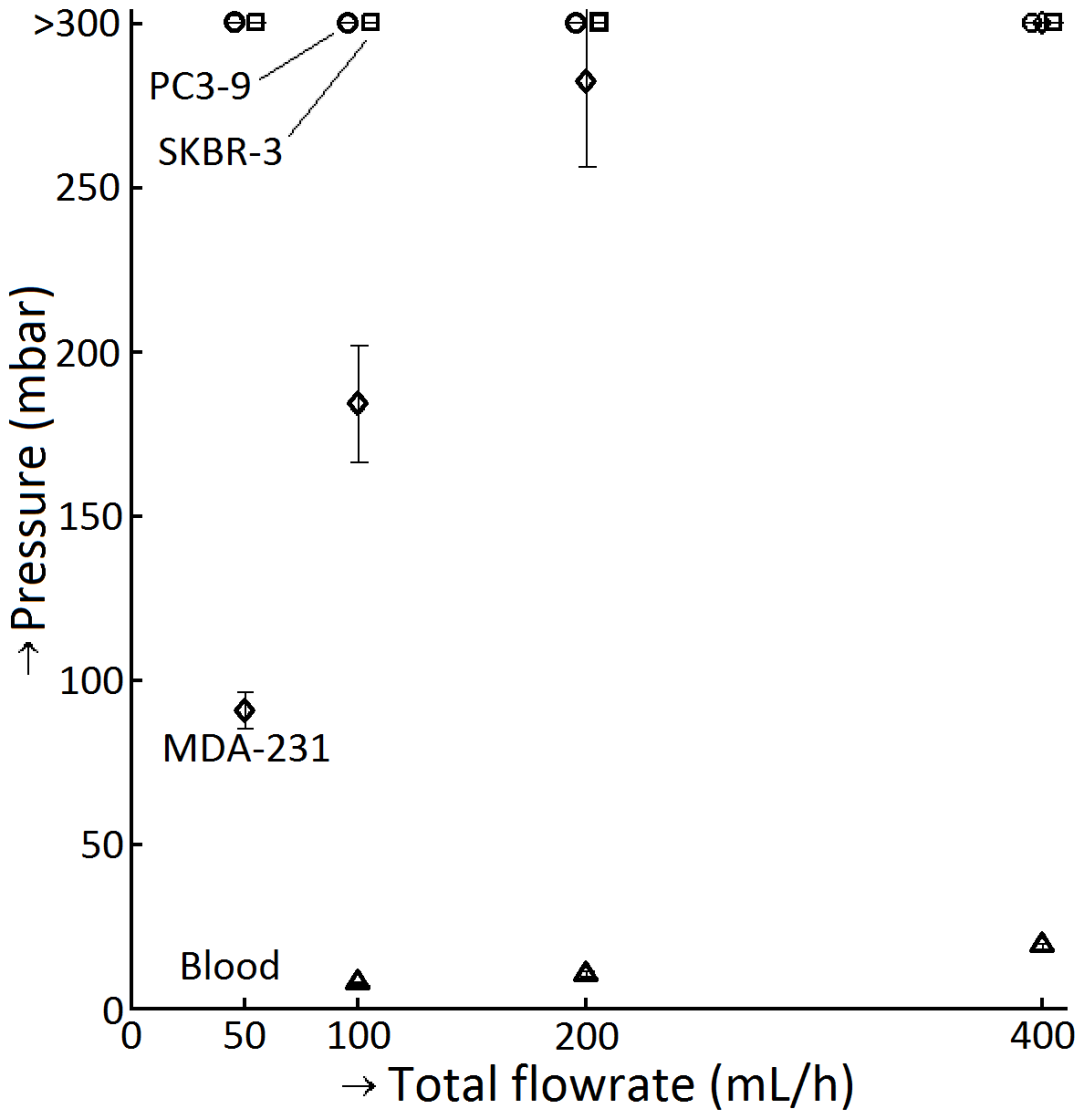
Culture cells can pass through small pores at low pressures. The y-axis shows pressure drops when 1.0·10^6^ SKBR3, PC3-9 or MDA-231 cell lines are filtered across 5 µm track-etched filters with 0.35·10^6^ pores. Data points were all measured at total flow rates of 50, 100, 200 and 400 mL/hr, but were slightly offset to facilitate reading of the graph. The median cell diameter of PC3-9 = 19.0 µm, SKBR-3 cells 16.4 µm and MDA-231 = 15.4 µm and were thus expected to occupy all pores. All samples with SKBR-3 and PC3-9 samples clogged the filter and the sample with MDA-231 clogged at a flow rate of 400 mL/h.

We filtered 10^6^ PC3-9, SKBR-3 and MDA-231 culture cells at different flow rates using 5 µm track-etched filters. We expected the filter to clog (pressure to rise above 300 mbar) before 0.35·10^6^ culture cells were passed through a track-etched filter with 0.35·10^6^ pores, as these cells are more than three times larger than the pore size. Both PC3-9 and SKBR-3 clogged at all flow speeds when 0.3·10^6^ up to 0.5·10^6^ cells had been injected. The MDA-231 cells only clogged the filter at the highest total flow speed of 400 mL/h, but 10^6^ cells passed the filter at total flow speeds of 50, 100 and 200 mL/h. Figure 6 shows the pressures and flow rates for the three cell lines, with each data point representing the average of triplicate measurements and error bars indicating the standard deviation. The implication of MDA-231 not clogging the filter was that a substantial fraction of these cells passed the 5 µm pores. Considering only the ratio of spiked cells to pores, at least 65% of MDA-231 cells passed the filter. The loss is likely to be higher because a single MDA-231 cell could block multiple pores. In addition, the PC3-9 and SKBR-3 runs with the lowest flow speed clogged when more than 40% of the sample had been injected, suggesting that also for these cell lines a substantial fraction of cells had passed the filter.

To verify the size selectivity of the 5 µm filter, the filtrate of a solution containing 10^6^ polystyrene beads with diameters of 4, 6, and 10 µm was investigated. We found that 26% of the 4 µm beads injected, 0.35% of the 6 and 0.0% of the 10 µm beads had passed the filter. The beads not found in the filtrate were found on the filter. It was possible to pass 15 µm MDA-231 cells through a pore of only 5 µm at relatively low pressures of 80 mbar, while 6 µm polystyrene beads were retained. This difference in behavior between beads and cells may be explained by a difference in stiffness. Therefore, the passage of a cell through a pore was modeled as a high viscosity stream passing through a filter.

### MDA-231 cells pass at least 4300 times slower through a 5 µm pore than a WBC

**Figure 7:**
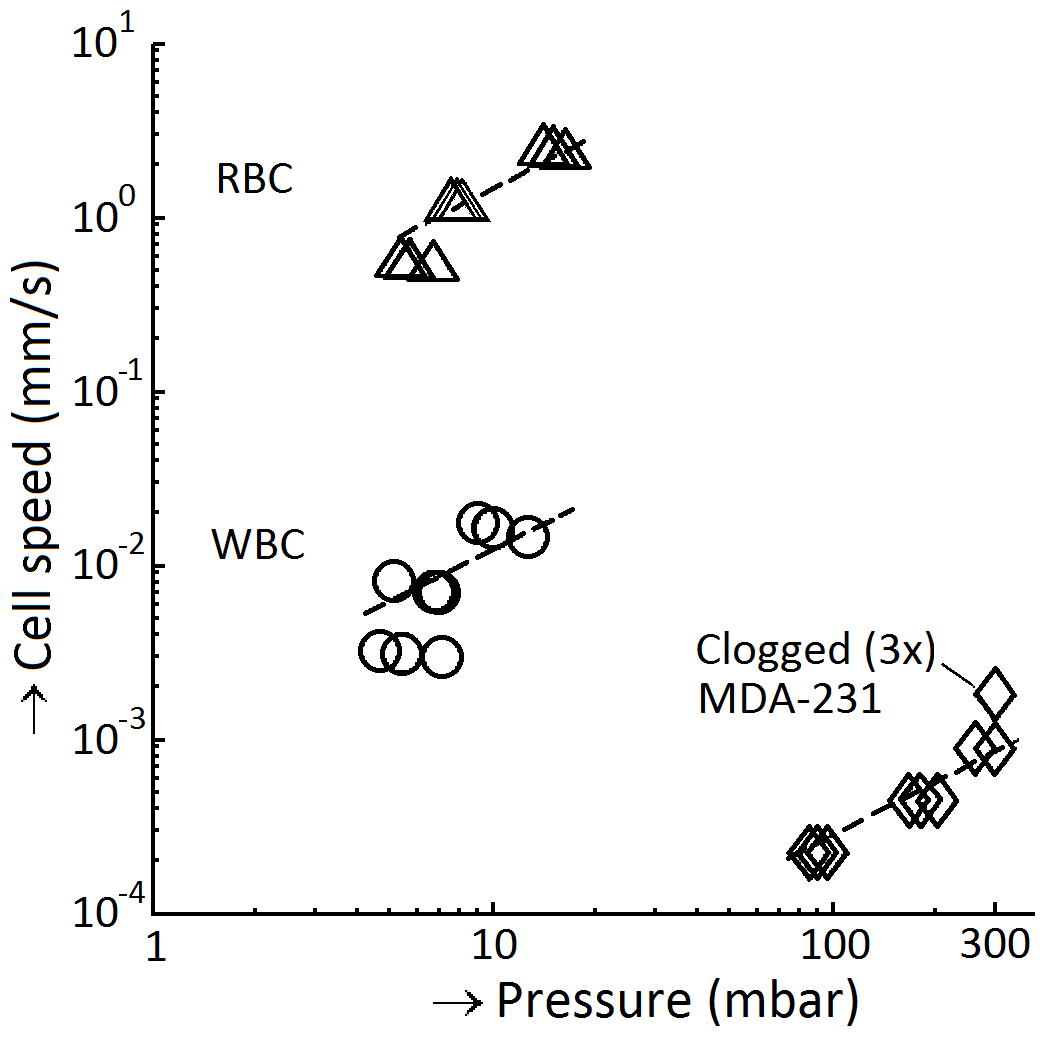
Estimated speeds for different cells. Estimated speed inside a filter pore versus pressure across the filter for white (WBC) and red blood cells (RBC) and MDA-231 culture cells. Dashed lines are fits of 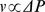. All three MDA-231 samples at a flow rate of 400 mL/h clogged.

The experimental setup and equation 3 were used to estimate the speed with which an MDA-231, WBC or RBC passes through a pore and derived from this the apparent viscosity of a cell relative to the pore, figure 7. Due to the large relative error in the pressures detected for the 8 µm filters we did not estimate the speed of passage for this pore size. From linear fits through the data, we find that at the same pressure, the passage of RBC is approximately 120-fold faster than the passage of WBC and MDA-231 cells are 430 times slower than the WBC. The high viscosity stream approximation was used to determine the apparent viscosity of each cell type from the slope between pressure and time of passage. The apparent viscosity for 5 µm pores is estimated at 0.035±0.010 Pa·s for RBC (ΔP = 5−16 mbar), 3.2±2.5 Pa·s for WBC (ΔP = 5−13 mbar), and of 1.6·10^3^±0.2·10^3^ Pa·s for MDA-231 cells (ΔP = 85−300 mbar). The SKBR-3 and PC3-9 cells clogged the filter even at the lowest speed, their apparent viscosity must be larger than 2·10^3^ Pa·s. Experiments with granulocytes suggest cells are shear thinning [39], [45], with apparent viscosity reducing from 2.4·10^3^–4.4·10^3^ Pa·s [46] at 0.2 mbar to 30–200 Pa·s at pressures near 5 mbar [39], [47]–[49]. If this holds true for cells derived from tumor cell lines, the time needed to pass a pore is even larger at lower pressures. The estimates for WBC and RBC apparent viscosity as determined here in bulk cell suspensions are similar to those determined using micropipette aspiration at similar pressures and pore sizes. We found 3.2 Pa·s for WBC, compared to 30–200 Pa·s for granulocytes reported in the literature [39], [47]–[49]. For RBC the apparent viscosity was determined to be 35·10^−3^ Pa·s and for MDA-231 cells we determined an apparent viscosity of 1.6·10^3^ Pa·s at a pressure of 100 mbar and greater than 2·10^3^ Pa·s for SKBR-3 and PC3-9 culture cells. At a pressure of 10 mbar, the speed of a RBC in a 5 µm pore is 1.5 mm/s, compared to 12·10^−3^ mm/s for a WBC. Literature values are comparable with WBC entry speed into a glass capillary of 36·10^−3^ mm/s [50] and a 700–1000 fold difference between passage time through a filter of RBC and WBC [51], [52]. At a pressure of 100 mbar an MDA-231 cell passes in 360 s. Assuming that the apparent viscosity is independent of pressure, at 10 mbar an MDA-231 cell would pass the filter in one hour.

An important difference between the apparent viscosities as determined by micropipette aspiration and those determined using a filter is that the entire cell can be aspirated into a capillary, while WBC and MDA-231 cells are larger than the pore; the pore volume is 35% of the WBC volume and 11% of the MDA-231 cell volume. As a result, the cells need to deform less to pass through a pore than to enter a long capillary, and thus have lower apparent viscosity. The relative difference between apparent viscosity of blood cells and tumor cells is important because it determines whether captured tumor cells will be on the filter at the end of filtration. It is unknown if these tumor cell lines are a good model for CTC, as very little is known about the physical properties of CTC. Many approaches to determine such properties, including the approach we applied, require large numbers of cells, while CTC in patients are extremely rare. Despite differences between our bulk method and micropipette experiments with similar pore size, the latter could be applied to determine the apparent viscosity of real CTC, an advantage since these require only 1 cell per determination. In addition, micropipette experiments could be used to determine the influence of various parameters such as pressure and sample temperature [39] on the apparent viscosities of blood cells and various cultured tumor cells.

Micropipette aspiration experiments have shown that a critical pressure exists for which a cell is not pulled into the pipette, due to surface tension of the cortex. For granulocytes this critical pressure is 0.1 mbar on a 5 µm pore [53], while for various cancer cell lines this pressure is 2–8 mbar [54]. The apparent viscosity is much higher when determined at a pressure slightly above the critical pressure. This suggests that, for a 5 µm pore size, the sample should be filtered at a pressure near 2 mbar in order to retain all captured culture cells. However filtration of 1 mL of blood at this pressure takes approximately 15 minutes on a 1 cm^2^ track etched filter with 5 µm pores, resulting in flow that is extremely slow. This leads to uneven sample distribution across the filter due to gravity, defeating the purpose. While it is not practical to filter at a pressure below the critical pressure, apparent viscosity is highest near the critical pressure, which suggests that filtration at pressures near the critical pressure of a CTC will yield better results than filtration at much higher pressures.

### Fixation dramatically increases the pressure needed to push cells through a pore

**Figure 8:**
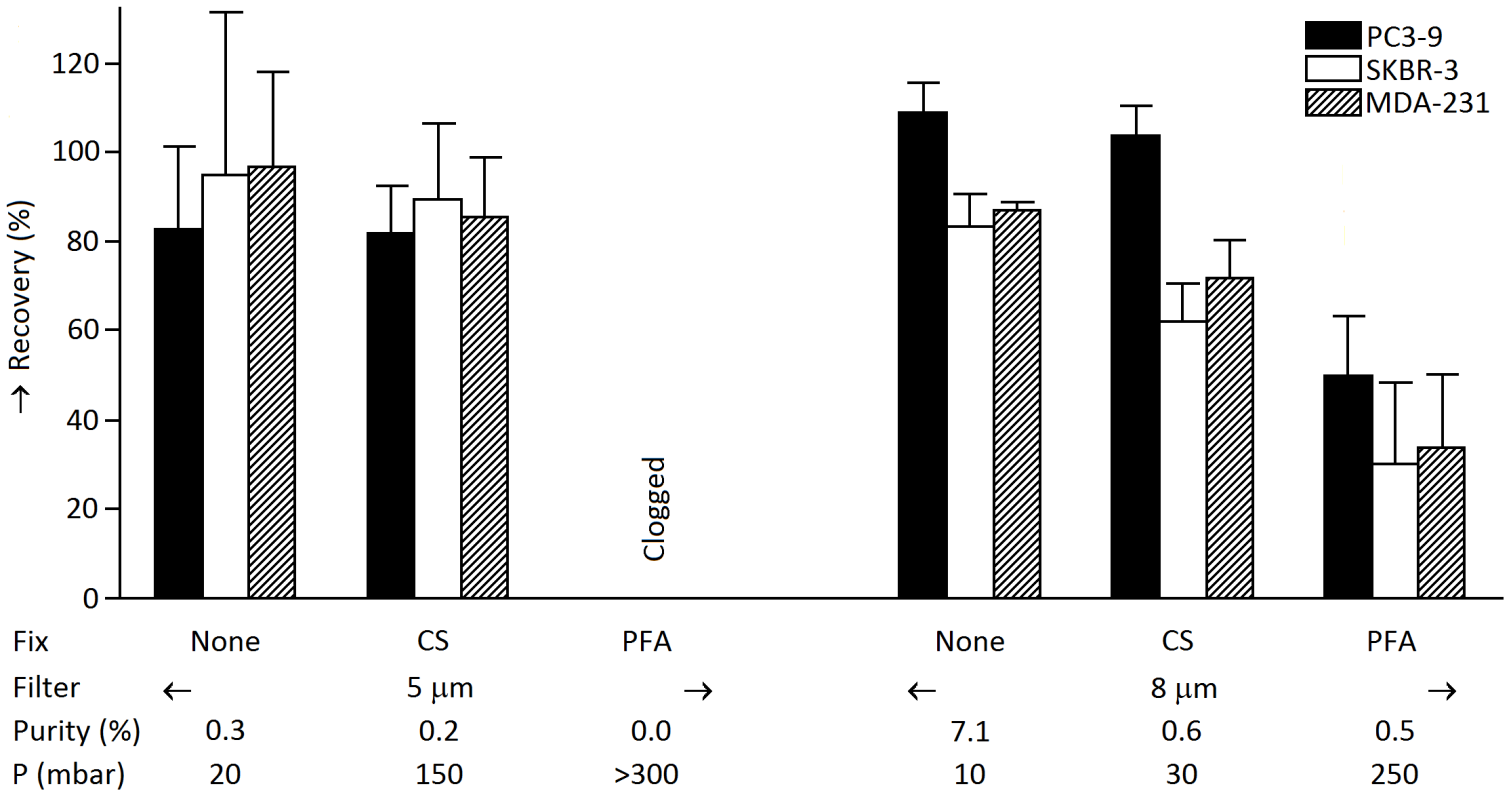
Influence of fixation on recovery, pressure across filter and sample purity. Bars show recovery of three culture cell lines spiked into 1 mL of whole blood as a function of fixation and pore size (5 µm and 8 µm). Samples were not fixed, fixed for 4 hours in CellSave (CS) or 10 minutes in 0.4% formaldehyde (PFA). Whiskers represent 1 standard deviation. Average sample purity is defined as the % cell line of total cells and is shown below the bar plot. Peak pressure (P) is shown below the bar plot as well. Recovery and purity are both highest for no fixation. The pressure needed to pass 1 mL of blood through the filter is much larger with fixed samples, with the pressure required to pass PFA fixed blood through the 5 µm in excess of 300 mbar that could be determined on our setup.

Fixation can be used to change the apparent viscosity, to investigate its influence the recovery of cultured tumor cells as a function of fixation was determined by spiking 300 cells of 3 different cell lines into 1 mL of blood. The recovery for each cell line, fixation type and pore size is shown in figure 8. Pressure across the filter and sample purity is shown below the x-axis. The purity was defined as the percentage of cancer cells of all cells recovered. Unfixed samples had highest recovery and purity at the lowest pressure. CellSave fixed samples had recovery slightly lower than unfixed samples, with 3–8 fold higher pressures, and lower purity. PFA fixed samples clogged the 5 µm filter. PFA fixed samples on the 8 µm filter had lower recovery and lower purity than the other samples, while the pressure was 25 fold higher compared to unfixed samples. Overall, the 5 µm and 8 µm track-etched filters with unfixed samples performed similar in terms of recovery but sample purity was an order of magnitude higher on the 8 µm track-etched filter. Fixation increases the apparent viscosity of a cell, which increases the pressure needed to pass a cell through the same pore size in the same time, or increases the pore size needed at constant pressure and time. The lower sample purity of PFA fixed samples compared to unfixed samples suggests that the difference in passage time and apparent viscosity between culture cells and white cells is reduced after fixation.

All other experiments presented here were performed with fresh unfixed blood. It is conceivable that processing of older samples requires some form of fixation for sample preservation, for example to allow processing in a remote laboratory. CellSave [55] is a gentle fixative used to preserve CTC in blood prior to immunomagnetic enrichment, and may prove to be a good preservative for enrichment of CTC prior to size based filtration.

### Filtration process

From the results reported in the literature and in this manuscript we interpret the filtration process as follows: cells and diluent arrive on the filter. Cells are pulled to a pore by fluid passing through that pore and proceed to slowly pass through, with cells with a higher apparent viscosity passing through slower, resulting in a relative enrichment of cells with higher apparent viscosity. A pore is occupied by a cell most of the time and passes diluent in short bursts between cells. If the pressure during filtration is lower than the critical pressure for only one cell type in the suspension, this cell type has an infinite apparent viscosity and will be retained. This is difficult to achieve in any practical setup, and pressure during filtration is most likely higher than the critical pressure for all cell types. Thus all cells types will eventually pass through the filter, resulting in reduced recovery of tumor cells from larger sample volumes (i.e. longer filtration). If possible the filtration time should be shorter than the time a tumor cell needs to pass the filter at the operating pressure so recovery is maximized. If this is not possible it could be mitigated by stacking multiple filters on top of each other, thus dramatically increasing the relative difference in passage time between tumor cells and blood cells. If cortical tension returns the cells to the top of the filter when no pressure difference exists, the same could be achieved by intermittent flow, passing blood cells in bursts and then waiting for the tumor cells to return to the top of the filter between bursts.

## Conclusions

While CTC enrichment by filtration has been first performed more than 50 years ago [18], [19], a systematic comparison of assay parameters was lacking to date. We conclude that the optimal conditions include a pressure close to or lower than 10 mbar for a pore size of 5 µm and no fixation.

Total processing time is limited because CTC still move slowly through the filter with a speed depending on the operating pressure; at a pressure of 10 mbar filtration should be completed in less than 1 hour for capture of MDA-231 cells. Sample dilution is not strictly needed, and has a small negative influence on recovery. If fixation is needed for sample preservation, a gentle fixative should be used, in which case the ideal filtration pressure will probably be higher.

To further optimize the conditions, the difference between the apparent viscosities of CTC and other blood component has to be maximized. Tumor cell lines are of limited use since their properties may not sufficiently resemble CTC. Since CTC are very rare, a purely empirical approach that requires large numbers of CTC has to be supplemented by other methods to determine the mechanical properties (surface tension, elasticity) of different types of CTC under various conditions (fixation, buffers, etc.) and predict the apparent viscosities as a function of pore size and pressure from these properties.
